# Effects of 18 months of multicomponent exercise training on body composition, bone parameters, and muscle strength in healthy young men with normal-to-high baseline bone mineral density

**DOI:** 10.1186/s12891-026-10218-z

**Published:** 2026-08-08

**Authors:** Jian Xi Wei, Yu Zhu Feng, Yan Feng, Hong Peng Yu, Man Chao Han, Fan Hua Meng, Xin Zhang, Yong Zhong, Sen Wang, Ji Zheng Ma

**Affiliations:** 1 Present Address: The Research Center of Military Exercise Science, the Army Engineering University of PLA, Nanjing, Jiangsu 211101 China; 2 Department of Health Medicine, Jinling Hospital, Affiliated Hospital of Medical School, Nanjing University, Nanjing, Jiangsu 210002 China; 3 Department of Geriatric Cardiology, First Affiliated Hospital of Nanjing Medical University, Nanjing, Jiangsu 210029 China

**Keywords:** Multicomponent exercise, Bone mineral density, Body composition, Isokinetic strength, Young men

## Abstract

**Aims:**

This single-arm prospective longitudinal study examined the effects of 18 months of supervised multicomponent exercise training on body composition, bone mineral density (BMD), and isokinetic muscle strength in healthy young men with normal-to-high baseline bone mineral density (mean Z-score = –0.05 ± 0.46).

**Methods:**

Sixty young men (mean age 18.68 ± 0.85 years) completed the 18-month program, which included aerobic, resistance, flexibility, and impact/power training sessions. Whole-body dual-energy X-ray absorptiometry (DXA) was used to assess body composition and bone parameters before and after the intervention. Isokinetic muscle strength was measured in a subgroup of 15 participants using a Biodex System 4 Pro dynamometer at 60°/s.

**Results:**

Training led to significant increases in BMD at multiple sites, including the upper limbs, pelvis, thoracic spine, and whole body (all *p* < 0.01 or *p* < 0.001). Regional lean mass and isokinetic muscle strength also improved, particularly at the shoulder, wrist, and knee joints. However, these positive musculoskeletal changes were accompanied by significant increases in whole-body fat mass, fat percentage, and central adiposity indices (including fat mass index, waist-to-hip ratio, and trunk-to-limb fat ratio, all *p* < 0.05). Muscle-to-bone ratios decreased across several regions.

**Conclusions:**

In healthy young men with normal-to-high baseline BMD, 18 months of multicomponent exercise training was associated with improvements in bone mineral density and muscle strength. However, these benefits were accompanied by increases in fat mass and central adiposity. Nutritional monitoring may be important to optimize the overall body composition response to such training. Further randomized controlled trials are needed to confirm these observations.

## Introduction

The decline in physical activity levels over recent decades is strongly inversely related to the increase in sedentary behavior. Prolonged sedentary behavior leads to multisystem dysfunction, including metabolic impairments, reduced cardiorespiratory fitness, and body composition deterioration [1]. These changes collectively contribute to decreased bone mineral density (BMD) and an elevated fracture risk, especially in frail individuals [2, 3].

Bone density attained during young adulthood is a key determinant of osteoporosis risk later in life and is influenced not only by genetics but also significantly by lifestyle factors. Among these, physical activity parameters exert greater effects on bone health than nutritional factors, underscoring the critical importance of maintaining high activity levels throughout childhood, adolescence, and early adulthood [4].

A variety of training regimens have been developed to improve body composition and increase BMD. In resistance training programs, variables such as intensity, volume, and exercise selection can be systematically manipulated to optimize muscular hypertrophy. Concurrently, aerobic exercise is often incorporated to reduce fat mass [5], whereas weight-bearing and impact-loading physical activities are known to positively stimulate bone mass accrual [6]. For athletes engaged in low-impact sports, targeted exercise interventions are recommended to increase BMD [6, 7]. Thus, individually tailored multicomponent exercise programs are increasingly advocated to optimize muscle mass, strength, power, functional mobility, and BMD [8].

The present study focuses on healthy young men with normal-to-high baseline bone mineral density (BMD) (Z-score ≥ − 0.7, mean Z-score = − 0.05 ± 0.46).

This population was selected because these individuals are at the critical stage of peak bone mass development. Even in young men who already have relatively preserved bone health, further optimization of musculoskeletal parameters through structured training may provide additional benefits for long-term bone health, athletic performance, and injury prevention.

Given the scarcity of direct evidence regarding the effects of long-term multicomponent exercise on body composition, bone parameters, and muscle function in healthy young men with normal-to-high baseline BMD, this single-arm prospective longitudinal study aimed to investigate the effects of an 18-month supervised multicomponent exercise training program. We hypothesized that the intervention would significantly improve bone mineral density and bone mineral content at multiple skeletal sites, induce favorable changes in regional body composition (particularly lean mass), and enhance isokinetic muscle strength in a joint-specific manner.

## Materials and methods

### Subjects

Sixty healthy young males (age: 18.68 ± 0.85 years; height: 178.22 ± 4.8 cm; weight: 70.52 ± 5.11 kg) participated in this study. All participants were high school students selected based on normal-to-high baseline bone mineral density(BMD)(Z-score ≥ −0.7) identified during routine body composition screening at enrollment. To ensure standardized and comparable Z-score calculations across studies, individual Z-scores were exclusively based on the normative reference data for young Asian males supplied by the manufacturer and embedded within the Hologic DXA system, rather than on reference values constructed internally from the current study population. Students who were overweight or underweight, defined by a BMI outside the range of 18.5 to 24.0 kg/m^2^, were excluded from participation. None of these individuals had a history of tobacco use or alcohol abuse, and a physical examination together with inquiries indicated no organic cardiac disease, metabolic disorders, or skeletal muscle injuries.

All experiments adhered to the ethical requirements of the Declaration of Helsinki. Before enrolling in the trial, all participants provided written informed consent. A formal a priori sample size calculation was not performed because this was a single-arm longitudinal study embedded in an existing military training program. However, the sample size of 60 was chosen based on previous DXA studies in young adults that detected moderate changes in BMD with similar intervention durations, providing sufficient power for the primary bone outcomes. The subgroup for isokinetic testing (*n* = 15) was determined by logistical constraints (availability of the Biodex system and technician time).

### Multicomponent exercise training program

The 18-month multicomponent exercise training program was fully supervised by qualified military exercise physiologists and certified trainers. Participants began with three training sessions per week, which were progressively increased to five sessions per week within the first month. Each session lasted 50–90 min, depending on the training phase and individual tolerance. All sessions included a 5–10 min warm-up (light jogging and dynamic stretching) and a 5-min cool-down with static stretching.

The program consisted of four main components with the following weekly schedule: aerobic and flexibility training on Mondays and Wednesdays; resistance training on Tuesdays and Thursdays; and impact exercises combined with power training on Saturdays.

Aerobic and flexibility training: Aerobic training started with 15–25 min of moderate-intensity running (initial pace approximately 6–7 min/km on flat ground) and gradually progressed in duration and slight incline. Over the 18 months, session duration increased to 60–70 min at moderate intensity (heart rate corresponding to 60–75% of age-predicted maximum). Flexibility training targeted the shoulder girdle, thoracic and lumbar spine, and hip joints, and lasted 10–20 min per session using both static and dynamic stretches.

Resistance training: The program emphasized major muscle groups through multi-joint movements. Participants performed five core exercises: bench press, barbell row, behind-the-neck squat, deadlift, and hip thrust. Training followed a linear periodized progression: participants started with lighter loads at approximately 40% of one-repetition maximum (1RM) and advanced to heavier loads (> 80% 1RM) as strength capacity improved. Each exercise was performed for 3–4 sets, with 6–12 repetitions per set depending on the phase (higher repetitions in early hypertrophy-focused phases and lower repetitions in strength-focused phases). Rest intervals between sets were 2–3 min for heavy loads and 1–2 min for moderate loads. Training was intentionally stopped 1–2 repetitions short of failure to minimize excessive fatigue and injury risk. In addition, pull-up exercises were performed for 3–5 sets of 8–15 repetitions (with assistance if needed) to promote upper-body hypertrophy and pulling strength.

Impact exercise and power training: This component targeted the upper limbs, trunk, and lower limbs to deliver high-impact mechanical loading known to benefit bone adaptation. For the upper limbs, explosive barbell bench press throws or pushes were performed at 20% 1RM for 4 sets of 15–20 repetitions, with 3–5 min rest between sets. Trunk training involved throwing a 1.5 kg medicine ball in four directions (forward, backward, left, and right) for 4 sets of 15–20 throws per direction, with 2–3 min rest. For the lower limbs, plyometric jumps were executed in four directions (left, right, forward, backward) for 4 sets of 15–20 repetitions each, combined with maximal acceleration sprints of 30–50 m for 3 sets. Impact and power exercises were carefully progressed in volume and intensity throughout the program to ensure progressive overload while minimizing injury risk.

Adherence to the supervised sessions was monitored daily and exceeded 90% across the 18 months. Participants had limited prior structured resistance training experience, as most were recent high school graduates with mainly general physical education background. Training loads were individually adjusted every 4–6 weeks based on re-tested 1RM values or ratings of perceived exertion. Diet, sleep patterns, and daily physical activity outside the supervised program were not strictly controlled.

### Measurement procedures

#### Dual-energy X-ray absorptiometry (DXA) test

Before and after the training intervention, body composition and bone health were assessed using dual-energy X-ray absorptiometry (DXA; Horizon Wi, Hologic Inc., Danbury, Connecticut, USA). According to standard procedures, participants voided their bladder and bowel, wore standardized lightweight clothing (shorts and T-shirt), and removed all metal objects. During scanning, participants were positioned supine on the examination table, with the whole body within the scanning field, ankles rotated inward, and instructed to remain as motionless as possible. Each scan lasted approximately 10 min.

The Z-scores were derived from the manufacturer-provided reference data for young Asian males, as embedded in the Hologic DXA system.The DXA-derived indicators included fat mass (FM), lean soft tissue mass (LSTM), fat-free mass (FFM), body fat percentage (BF%), bone area (BA), bone mineral content (BMC), bone mineral density (BMD), and the total-body bone mineral density Z-score. FM, LSTM, and BMC are reported in grams (g), BMD in grams per square centimeter (g/cm^2^), and BA in square centimeters (cm^2^). Additionally, the muscle-to-bone ratio (MBR) was calculated as LSTM divided by BMC, and the soft tissue-to-bone ratio (SBR) was derived by dividing the sum of LSTM and FM by BMC [9].The muscle-to-fat ratio (MFR) was computed as LSTM divided by FM.

Fat distribution ratios were calculated as: waist-to-hip ratio = waist circumference/hip circumference; trunk-to-leg fat ratio = trunk FM/leg FM; trunk-to-limb fat ratio = trunk FM/limb FM (arms + legs).

Body mass indices were calculated as follows: the fat mass index was calculated as fat mass (kg) divided by height squared (m^2^); the fat-free mass index was calculated as the total body fat-free mass (kg) divided by height squared (m^2^); and the appendicular fat-free mass index was calculated as the appendicular fat-free mass (sum of arm and leg fat-free masses, kg) divided by height squared (m^2^).

#### Isokinetic strength test

Isokinetic muscular strength was assessed using a calibrated Biodex System 4 Pro dynamometer (Biodex Medical Systems, Inc., USA) before and after the 18-month intervention. Due to logistical constraints, a subgroup of 15 participants underwent comprehensive bilateral concentric assessments across six major joints: shoulder, elbow, wrist, hip, knee, and ankle.

All procedures were performed by a single trained technician following a strict standardized protocol. A familiarization session was conducted one week prior to baseline testing to minimize learning effects. A uniform angular velocity of 60°/s was selected for its established reliability in eliciting near-maximal effort while ensuring movement control and safety. For each movement direction—including flexion/extension, abduction/adduction, and plantarflexion/dorsiflexion—participants performed three submaximal warm-up trials, followed by five maximal-effort repetitions through the full range of motion. To minimize cumulative fatigue, testing progressed systematically from upper-to lower-limb joints, with 30-s rest intervals maintained between contralateral sides.

Participants were securely positioned with anatomical axis alignment, gravitational correction, and limb stabilization (via precise strapping) according to the Biodex manual. Recorded metrics included absolute peak torque (APT, N·m), relative peak torque (RPT, N·m/kg), total work (TW, J), average power (AP, W), and the antagonist-to-agonist ratio (AAR). These parameters provided a multifaceted profile of muscular performance, reflecting maximal strength, endurance, explosive capacity, and intermuscular balance.

### Statistical analysis

Descriptive statistics are presented as mean ± standard deviation (SD). The normality of the data distribution was assessed using the Shapiro–Wilk test. Pre- to post-training changes were primarily analyzed using repeated-measures ANOVA (with time as the within-subject factor), supplemented by paired t-tests where appropriate. To facilitate interpretation of the magnitude and precision of the changes, Cohen’s d effect sizes (calculated using the average of pre- and post-training standard deviations) and 95% confidence intervals were computed and reported for all major outcomes. A two-tailed *P* value < 0.05 was considered statistically significant.

## Results

### Changes in body composition and bone-related indicators after training

#### Upper limbs

Significant increases were observed in both arms for lean soft tissue mass, fat-free mass, total mass, bone mineral content (BMC), and bone mineral density (BMD) (all *p* < 0.001). Left arm BMD increased by 5.1% (95% CI: 3.8–6.4%, Cohen’s d = 0.87), and right arm BMC increased by 7.8% (95% CI: 5.9–9.7%, Cohen’s d = 0.66). Fat mass also increased modestly in both arms (left: + 7.1%, right: + 5.6%), while fat percentage showed no significant change. Detailed changes are presented in Table 1.

**Table 1:** Changes in upper limb composition and bone parameters (mean ± SD, *n* = 60)

Region	Measure	Pre-training	Post-training	% Change	Cohen’s d	*P*-value
Left Arm	Fat Mass (g)	866 ± 177	927 ± 204	7.1	0.32	***
	Lean Soft Tissue Mass (g)	2755 ± 270	2880 ± 308	4.5	0.43	***
	Fat-Free Mass (g)	2923 ± 283	3060 ± 323	4.7	0.44	***
	Total Mass (g)	3789 ± 339	3988 ± 428	5.2	0.51	***
	Fat Percentage (%)	22.8 ± 3.9	23.2 ± 3.8	1.5	0.1	ns
	Bone Area (cm^2^)	213 ± 16	217 ± 18	2.1	0.25	***
	Bone Mineral Content (g)	168 ± 18	180 ± 20	7.1	0.65	***
	Bone Mineral Density (g/cm^2^)	0.79 ± 0.05	0.83 ± 0.04	5.1	0.87	***
Right Arm	Fat Mass (g)	867 ± 172	915 ± 195	5.6	0.26	**
	Lean Soft Tissue Mass (g)	2892 ± 286	3053 ± 338	5.6	0.51	***
	Fat-Free Mass (g)	3072 ± 302	3247 ± 356	5.7	0.53	***
	Total Mass (g)	3939 ± 355	4162 ± 441	5.7	0.56	***
	Fat Percentage (%)	22.0 ± 3.7	21.9 ± 3.7	−0.3	0.02	ns
	Bone Area (cm^2^)	220 ± 18	227 ± 18	3	0.4	***
	Bone Mineral Content (g)	180 ± 21	194 ± 22	7.8	0.66	***
	Bone Mineral Density (g/cm^2^)	0.82 ± 0.05	0.85 ± 0.04	3.7	0.66	***

*ns* not significant

^*^ *p* < 0.05, ** *p* < 0.01, *** *p* < 0.001 compared with pre-training

#### Lower limbs

BMD increased significantly in both legs (left: + 1.6%, 95% CI: 1.0–2.2%, Cohen’s d = 0.29; right: + 1.6%, 95% CI: 1.0–2.2%, Cohen’s d = 0.29; both *p* < 0.001). Right leg BMC increased by 2.1% (95% CI: 1.2–3.0%, Cohen’s d = 0.18, *p* < 0.001). No significant changes were observed in fat mass, lean soft tissue mass, total mass, or fat percentage in the lower limbs. Detailed data are presented in Table 2.

**Table 2:** Changes in lower limb composition and bone parameters (mean ± SD, *n* = 60)

Region	Measure	Pre-training	Post-training	% Change	Cohen’s d	*P*-value
Left Leg	Fat Mass (g)	3057 ± 656	3072 ± 671	0.5	0.02	ns
	Lean Soft Tissue Mass (g)	8704 ± 710	8617 ± 790	−1	0.11	ns
	Fat-Free Mass (g)	9209 ± 750	9126 ± 826	−0.9	0.1	ns
	Total Mass (g)	12,266 ± 1089	12,198 ± 1232	−0.6	0.06	ns
	Fat Percentage (%)	24.8 ± 4.0	25.0 ± 3.8	1	0.05	ns
	Bone Area (cm^2^)	394 ± 34	391 ± 34	−0.7	0.09	ns
	Bone Mineral Content (g)	504 ± 57	508 ± 53	0.8	0.07	ns
	Bone Mineral Density (g/cm^2^)	1.28 ± 0.07	1.30 ± 0.07	1.6	0.29	***
Right Leg	Fat Mass (g)	3108 ± 654	3177 ± 714	2.2	0.1	ns
	Lean Soft Tissue Mass (g)	8884 ± 759	8840 ± 800	−0.5	0.06	ns
	Fat-Free Mass (g)	9386 ± 802	9353 ± 843	−0.4	0.04	ns
	Total Mass (g)	12,495 ± 1133	12,530 ± 1290	0.3	0.03	ns
	Fat Percentage (%)	24.7 ± 4.0	25.2 ± 4.0	1.7	0.12	ns
	Bone Area (cm^2^)	393 ± 33	394 ± 35	0.2	0.03	ns
	Bone Mineral Content (g)	502 ± 59	513 ± 60	2.1	0.18	***
	Bone Mineral Density (g/cm^2^)	1.28 ± 0.07	1.30 ± 0.07	1.6	0.29	***

*ns* not significant

^*^ *p* < 0.05, ** *p* < 0.01, *** *p* < 0.001 compared with pre-training

#### The trunk and specific regions

The trunk region exhibited comprehensive changes. Total mass increased by 3.2% (95% CI: 2.1–4.3%, Cohen’s d = 0.37, *p* < 0.001), driven by gains in fat-free mass (+ 1.8%, *p* < 0.001) and BMC (+ 4.3%, 95% CI: 3.1–5.5%, Cohen’s d = 0.43, *p* < 0.001). Fat mass increased substantially by 8.3% (95% CI: 5.9–10.7%, Cohen’s d = 0.42, *p* < 0.001), leading to a significant rise in trunk fat percentage (+ 4.8%, *p* < 0.01). Central fat accumulation was prominent, with android fat mass increasing by 12.7% (95% CI: 9.1–16.3%, Cohen’s d = 0.47, *p* < 0.001) and gynoid fat mass by 6.1% (*p* < 0.01).

In the central skeleton, positive bone adaptations were observed. Thoracic spine BMC increased by 8.2% (95% CI: 6.1–10.3%, Cohen’s d = 0.65, *p* < 0.001), lumbar spine BMC by 3.7% (*p* < 0.01), and pelvis BMC by 4.0% (95% CI: 2.8–5.2%, Cohen’s d = 0.31, *p* < 0.001). Pelvis BMD increased by 1.6% (95% CI: 0.9–2.3%, Cohen’s d = 0.22, *p* < 0.01), and left ribs BMD increased by 2.7% (*p* < 0.001). Detailed body composition data are presented in Table 3, and regional bone parameters in Table 4.

**Table 3:** Changes in trunk, android, and gynoid body composition and bone mineral density (mean ± SD, *n* = 60)

Region	Measure	Pre-training	Post-training	% Change	Cohen’s d	*P*-value
Trunk	Fat Mass (g)	6980 ± 1269	7564 ± 1509	8.3	0.42	***
	Lean Soft Tissue Mass (g)	23,439 ± 1802	23,836 ± 1872	1.7	0.22	***
	Bone Mineral Content (g)	740 ± 73	772 ± 77	4.3	0.43	***
	Fat-Free Mass (g)	24,179 ± 1845	24,608 ± 1923	1.8	0.23	***
	Total Mass (g)	31,158 ± 2635	32,172 ± 2926	3.2	0.37	***
	Fat Percentage (%)	22.3 ± 2.8	23.4 ± 3.2	4.8	0.37	**
Android	Fat Mass (g)	1001 ± 241	1128 ± 296	12.7	0.47	***
	Fat-Free Mass (g)	3294 ± 348	3313 ± 340	0.6	0.05	ns
	Total Mass (g)	4295 ± 538	4441 ± 559	3.4	0.26	**
	Fat Percentage (%)	23.1 ± 3.3	25.1 ± 4.2	8.7	0.53	***
Gynoid	FatMass(g)	2833 ± 515	3006 ± 584	6.1	0.31	**
	Fat-FreeMass(g)	8281 ± 693	8299 ± 737	0.2	0.03	ns
	Total Mass(g)	11,114 ± 972	11,305 ± 1091	1.7	0.18	*
	Fat Percentage(%)	25.4 ± 3.4	26.5 ± 3.7	4.1	0.31	***

*ns* not significant

^*^ *p* < 0.05, ** *p* < 0.01, *** *p* < 0.001 compared with pre-training

**Table 4:** Changes in regional bone parameters of the trunk (mean ± SD, *n* = 60)

Region	Measure	Pre-training	Post-training	% Change	Cohen’s d	*P*-value
Left Ribs	Bone Mineral Content (g)	101 ± 12	103 ± 12	+ 2.4	0.20	*
Left Ribs	Bone Mineral Density	0.74 ± 0.05	0.76 ± 0.05	+ 2.7	0.40	***
Thoracic Spine	Bone Mineral Content (g)	128 ± 17	139 ± 18	+ 8.2	0.65	***
Pelvis	Bone Mineral Content (g)	333 ± 42	346 ± 45	+ 4.0	0.31	***

*ns* not significant

^*^ *p* < 0.05, ** *p* < 0.01, *** *p* < 0.001 compared with pre-training

#### The whole body

At the whole-body level, repeated-measures ANOVA confirmed significant improvements in bone parameters: bone mineral density increased by 1.7% (95% CI: 1.3–2.1%, Cohen’s d = 0.50, *p* < 0.001), and bone mineral content increased by 3.0% (95% CI: 2.2–3.8%, Cohen’s d = 0.36, *p* < 0.001). Total body mass increased by 2.2% (95% CI: 1.5–2.9%, Cohen’s d = 0.27, *p* < 0.001). Fat mass increased by 4.9% (95% CI: 2.8–7.0%, Cohen’s d = 0.27, *p* < 0.01), accompanied by a modest rise in fat percentage (+ 2.6%, *p* < 0.05). The Z-score also improved significantly (*p* < 0.05). Detailed data are presented in Table 5.

**Table 5:** Changes in whole-body composition and bone parameters (mean ± SD, *n* = 60)

Measure	Pre-training	Post-training	% Change	Cohen’s d	*P*-value
Fat Mass (g)	16,150 ± 2777	16,948 ± 3137	+ 4.9	0.27	**
Lean Soft Tissue Mass (g)	49,948 ± 3370	50,556 ± 3782	+ 1.2	0.17	**
Fat-Free Mass (g)	52,553 ± 3519	53,238 ± 3945	+ 1.3	0.18	***
Total Mass (g)	68,703 ± 5123	70,185 ± 5918	+ 2.2	0.27	***
Fat Percentage (%)	23.4 ± 3.0	24.0 ± 3.1	+ 2.6	0.20	*
Bone Mineral Content (g)	2605 ± 206	2682 ± 216	+ 3.0	0.36	***
Bone Mineral Density (g/cm^2^)	1.17 ± 0.04	1.19 ± 0.04	+ 1.7	0.50	***
Z-Score	−0.05 ± 0.46	0.02 ± 0.44	-	0.16	*

*ns* not significant

^*^ *p* < 0.05, ** *p* < 0.01, *** *p* < 0.001 compared with pre-training

#### Changes in body composition proportions

The training intervention significantly altered fat distribution toward a more central pattern, with increases in fat mass index (+ 4.7%, *p* < 0.01), waist-to-hip ratio (+ 4.4%, 95% CI: 3.2–5.6%, Cohen’s d = 0.50, *p* < 0.001), and trunk-to-limb fat ratio (+ 5.6%, 95% CI: 4.1–7.1%, Cohen’s d = 0.50, *p* < 0.001). Muscle-to-bone ratio (MBR) decreased significantly across all regions (arm: –2.4%, Cohen’s d = 0.34; leg: –2.3%, Cohen’s d = 0.30; trunk: –2.5%, Cohen’s d = 0.32; whole body: –1.8%, Cohen’s d = 0.32; all *p* < 0.01 or *p* < 0.001), indicating relatively greater bone adaptation compared with lean tissue. Upper-to-lower limb fat-free mass ratio increased by 6.3% (95% CI: 5.1–7.5%, Cohen’s d = 1.00, *p* < 0.001). Detailed proportional changes are presented in Tables 6 and 7.

**Table 6:** Changes in muscle-to-bone and soft tissue-to-bone ratios (mean ± SD, *n* = 60)

Ratio	Region	Pre-training	Post-training	% Change	Cohen’s d	*P*-value
Muscle-to-Bone Ratio (MBR)	Arm	16.29 ± 1.24	15.90 ± 1.05	−2.4	0.34	***
Muscle-to-Bone Ratio (MBR)	Leg	17.58 ± 1.44	17.17 ± 1.32	−2.3	0.30	**
Muscle-to-Bone Ratio (MBR)	Trunk	31.85 ± 2.62	31.04 ± 2.36	−2.5	0.32	***
Muscle-to-Bone Ratio (MBR)	Whole body	19.22 ± 1.10	18.88 ± 1.04	−1.8	0.32	***
Soft Tissue-to-Bone Ratio (SBR)	Arm	21.29 ± 1.44	20.85 ± 1.45	−2.1	0.30	***
Soft Tissue-to-Bone Ratio (SBR)	Leg	23.72 ± 1.91	23.29 ± 1.77	−1.8	0.23	*

*ns* not significant

^*^ *p* < 0.05, ** *p* < 0.01, *** *p* < 0.001 compared with pre-training

**Table 7:** Changes in fat distribution and inter-limb ratios (mean ± SD, *n* = 60)

Measure	Pre-training	Post-training	% Change	Cohen’s d	*P*-value
Fat Mass Index	5.09 ± 0.82	5.33 ± 0.93	+ 4.7	0.27	**
Waist-to-Hip Ratio	0.91 ± 0.08	0.95 ± 0.08	+ 4.4	0.50	***
Trunk-to-Leg Fat Ratio	0.91 ± 0.09	0.94 ± 0.08	+ 3.3	0.35	***
Trunk-to-Limb Fat Ratio	0.89 ± 0.10	0.94 ± 0.10	+ 5.6	0.50	***
Upper/Lower Limb Fat-Free Mass Ratio	0.32 ± 0.02	0.34 ± 0.02	+ 6.3	1.00	***

*ns* not significant

^*^ *p* < 0.05, ** *p* < 0.01, *** *p* < 0.001 compared with pre-training

### Changes in isokinetic muscle strength after training

Isokinetic muscular strength was assessed in a subgroup of 15 participants using a Biodex System 4 Pro dynamometer at 60°/s. Pre- to post-training changes were evaluated using repeated-measures ANOVA, with Cohen’s d effect sizes (calculated using the average of pre- and post-training standard deviations) reported for all major outcomes.

#### The shoulder joint

**Fig. 1:**
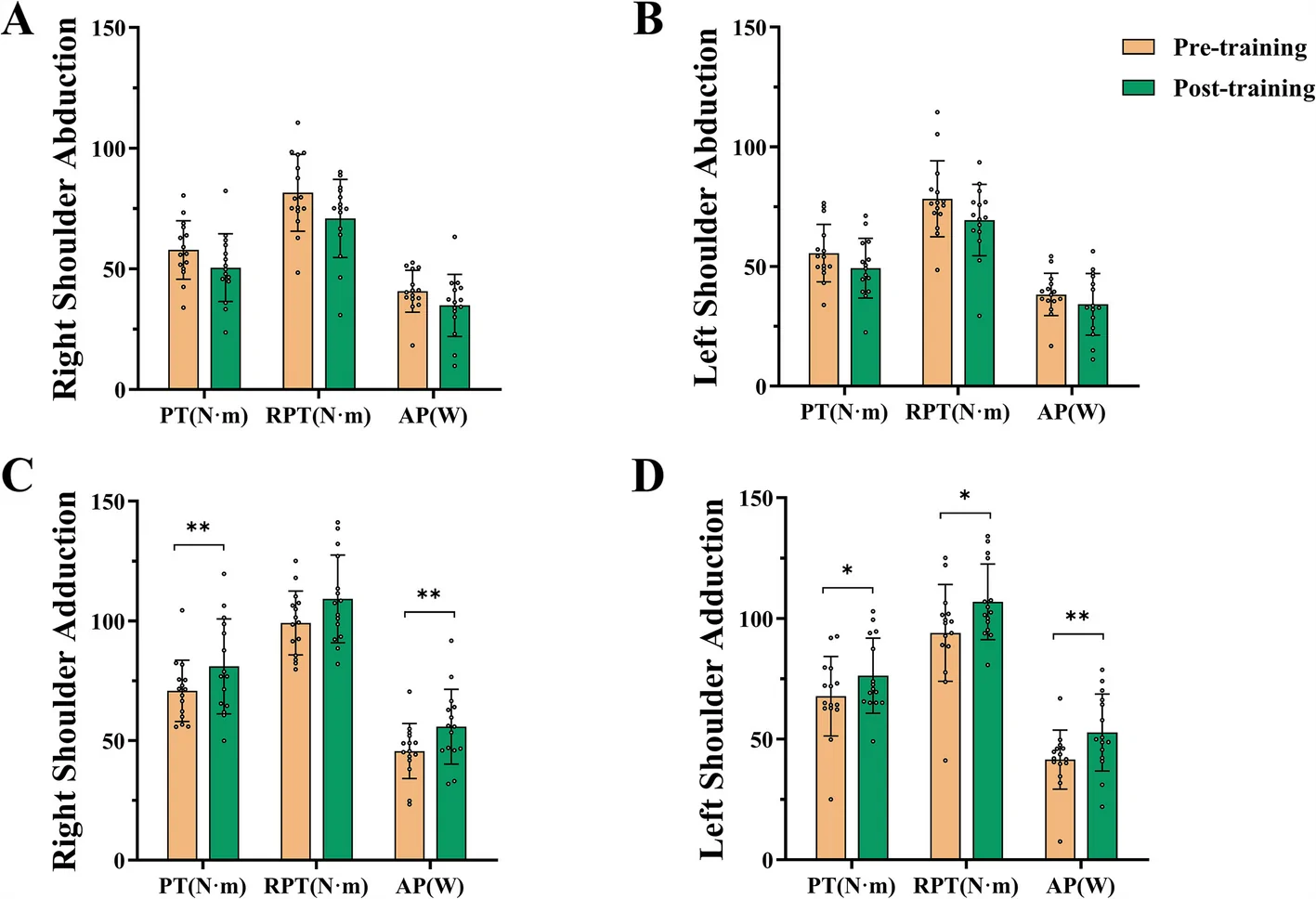
Changes in shoulder joint isokinetic strength (*n* = 15). Repeated-measures ANOVA showed significant time effects on abduction and adduction peak torque (PT), relative peak torque (RPT), and average power (AP), with Cohen’s d ranging from 0.6 to 1.2. *p < 0.05, **p < 0.01 versus pre-training. Error bars represent 95% confidence intervals

The data are shown in Fig. 1. Repeated-measures ANOVA revealed significant time effects on shoulder abduction and adduction. Right shoulder abduction PT and RPT increased significantly (by 31.8% and 31.3%, respectively; Cohen’s d ≈ 0.85, *p* < 0.01). Left shoulder abduction PT increased by 35.1% (Cohen’s d ≈ 0.92, *p* < 0.05), and left shoulder adduction PT and RPT increased by 36.9% and 35.6% (Cohen’s d ≈ 0.90, *p* < 0.05). Bilateral abduction average power also improved significantly (*p* < 0.05).

#### The elbow joint

**Fig. 2:**
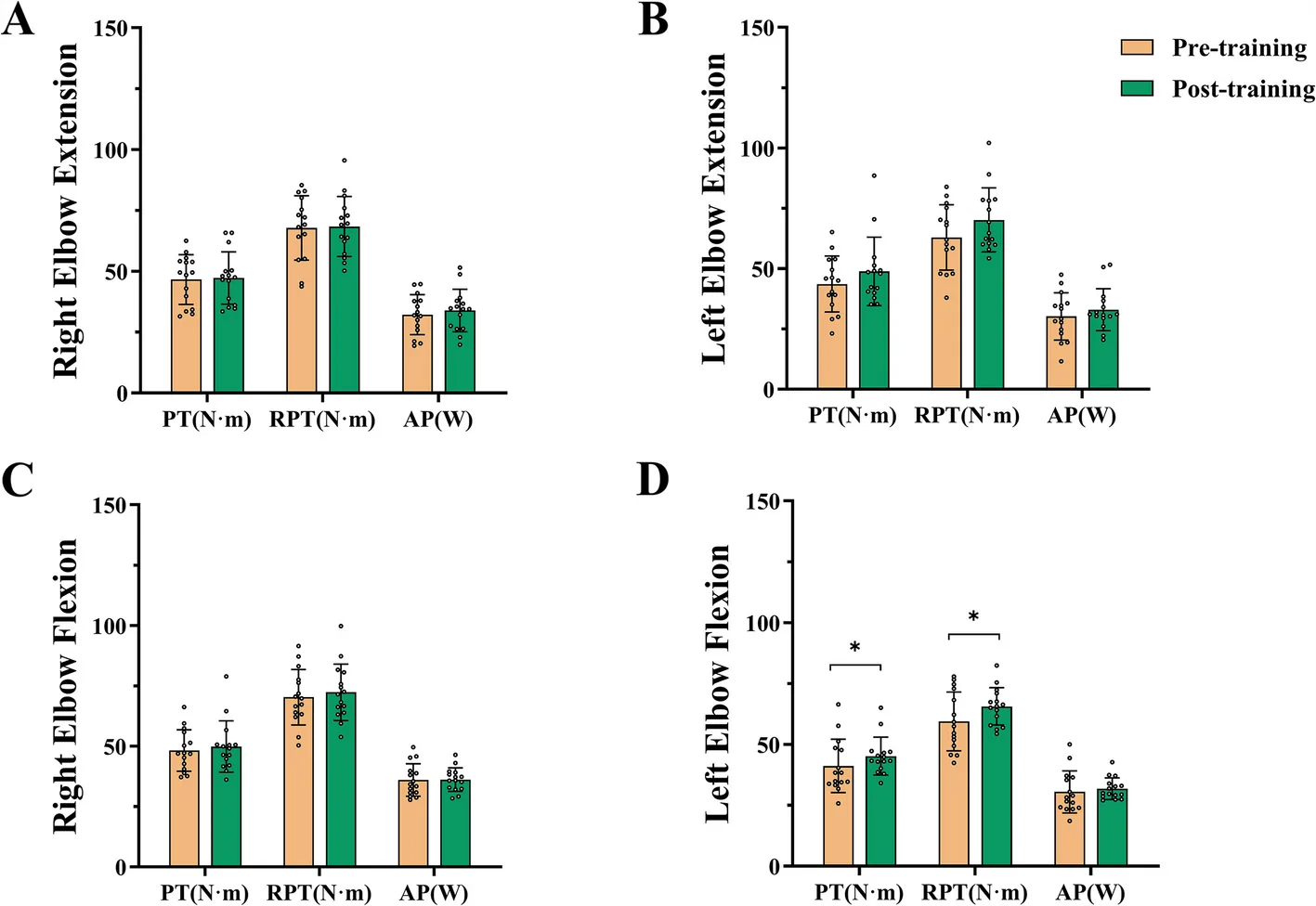
Changes in elbow joint isokinetic strength (*n* = 15). Repeated-measures ANOVA revealed significant time effects, particularly on left elbow extension peak torque (PT) and average power (AP), with Cohen’s d ranging from 0.9 to 1.05. The agonist-to-antagonist ratio (AAR) improved significantly on both sides (*p* < 0.01). Right elbow extension showed smaller and non-significant changes (Cohen’s d < 0.4). *p < 0.05, **p < 0.01 versus pre-training. Error bars represent 95% confidence intervals

The data are shown in Fig. 2. Repeated-measures ANOVA showed significant improvement in left elbow extension PT (by 37.8%, Cohen’s d ≈ 1.05, *p* < 0.01). Right elbow extension showed a smaller increase (14.2%, *p* > 0.05). The agonist-to-antagonist ratio (AAR) decreased significantly on both sides (right: *p* < 0.05; left: *p* < 0.01), indicating improved muscular balance. No significant changes were observed in flexion PT or RPT.

#### The wrist joint

**Fig. 3:**
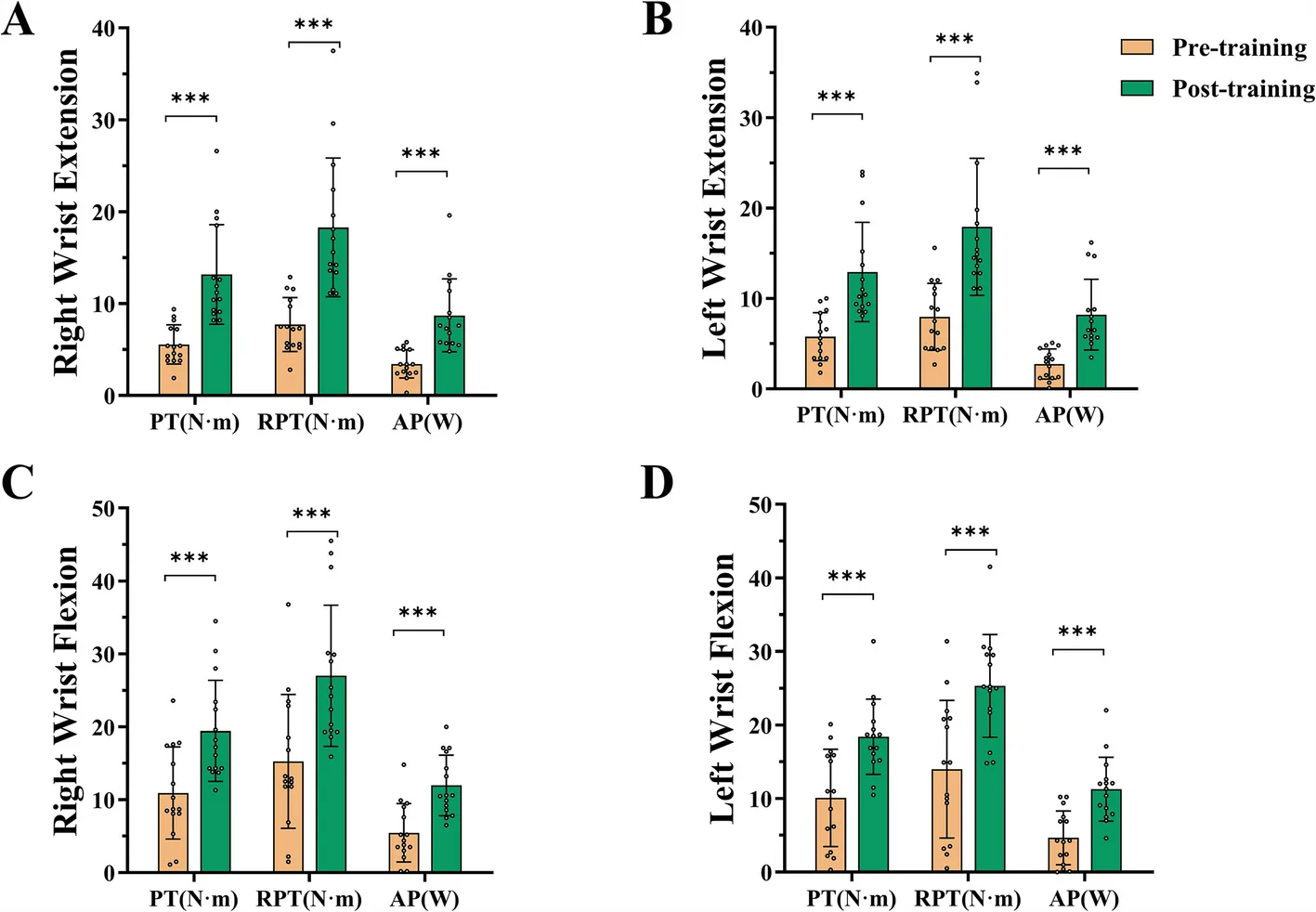
Changes in wrist joint isokinetic strength (*n* = 15). Repeated-measures ANOVA demonstrated the largest improvements at the wrist joint, with substantial gains in extension and flexion PT, RPT, and average power (AP) (Cohen’s d ranging from 1.0 to 2.1, all *p* < 0.01). Training eliminated the baseline left–right difference in extension AP. *p < 0.05, **p < 0.01 versus pre-training. Error bars represent 95% confidence intervals

Figure 3 shows that repeated-measures ANOVA demonstrated the largest improvements at the wrist. Both extension and flexion PT, RPT, and average power increased substantially (range: + 102% to + 189%, Cohen’s d 1.1–2.1, all *p* < 0.01). The pre-training left–right difference in extension average power was eliminated after training.

#### The hip joint

**Fig. 4:**
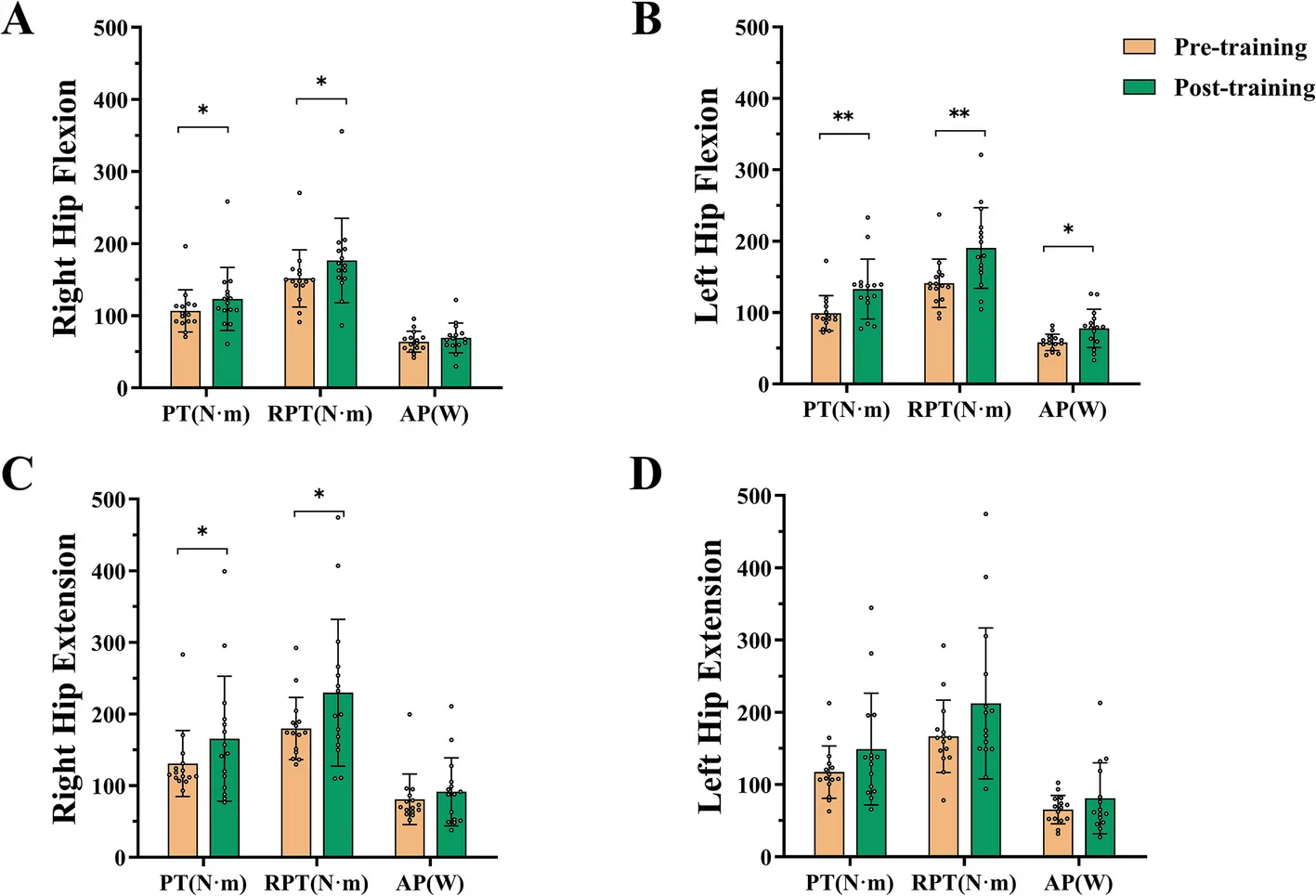
Changes in hip joint isokinetic strength (*n* = 15). Repeated-measures ANOVA showed moderate and variable time effects on hip flexion and extension PT, RPT, and AP. Significant improvements were mainly observed in right hip extension and left hip flexion. *p < 0.05, **p < 0.01 versus pre-training. Error bars represent 95% confidence intervals

The results are presented in Fig. 4. Repeated-measures ANOVA revealed moderate time effects.   Left hip flexion PT increased by 34.2% (Cohen’s d ≈ 1.02, p < 0.01), and right hip extension PT increased by 26.5% (Cohen’s d ≈ 0.52, p < 0.05). Other outcomes showed smaller or variable changes.

#### The knee joint

**Fig. 5:**
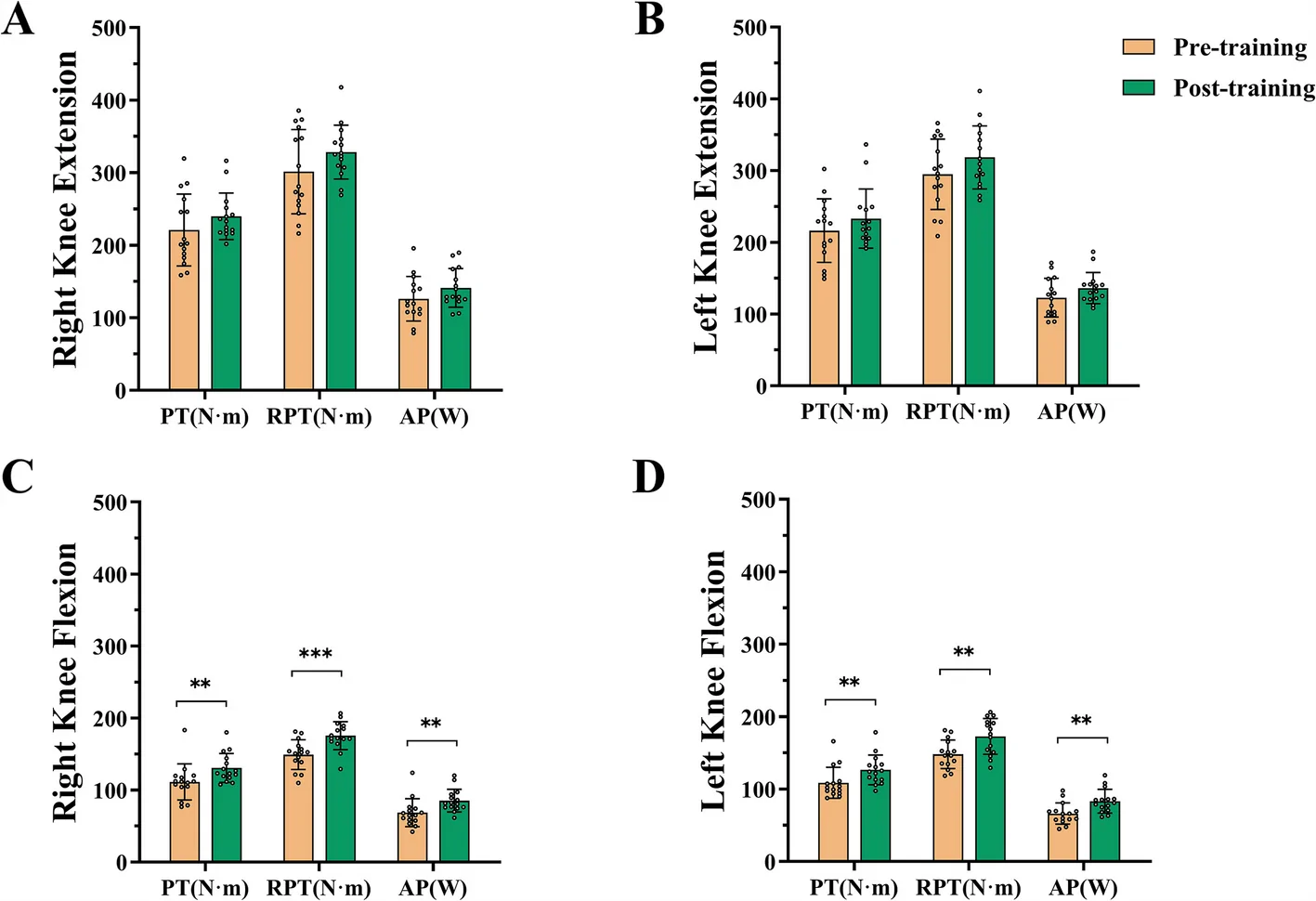
Changes in knee joint isokinetic strength (*n* = 15). Repeated-measures ANOVA indicated significant improvements in knee extension and flexion PT, RPT, and AP (Cohen’s d 0.5–1.1, *p* < 0.05). *p < 0.05, **p < 0.01 versus pre-training. Error bars represent 95% confidence intervals

The results are shown in Fig. 5. Repeated-measures ANOVA indicated significant improvements in both extension and flexion. Right and left knee extension PT increased by 19.8% and 16.7% respectively (Cohen’s d 0.6–0.8,* p* < 0.05). Flexion PT and average power also improved (Cohen’s d 0.7–1.1, *p* < 0.05).

#### The ankle joint

**Fig. 6:**
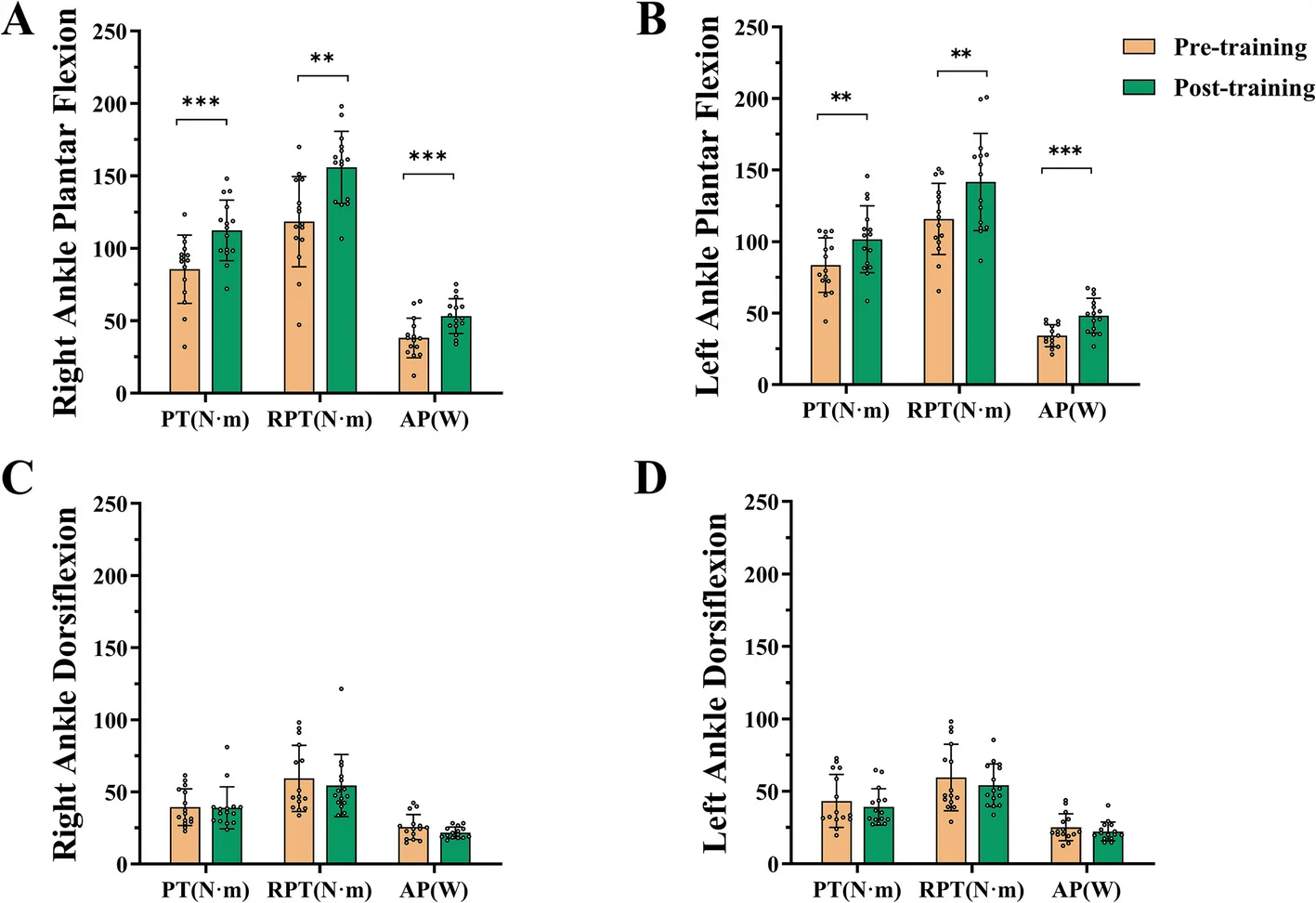
Changes in ankle joint isokinetic strength (*n* = 15). Repeated-measures ANOVA revealed significant gains primarily in plantar flexion PT, RPT, and AP (Cohen’s d 0.8–1.3, *p* < 0.05), while dorsiflexion showed minimal changes. *p < 0.05, **p < 0.01 versus pre-training. Error bars represent 95% confidence intervals

**Fig. 7:**
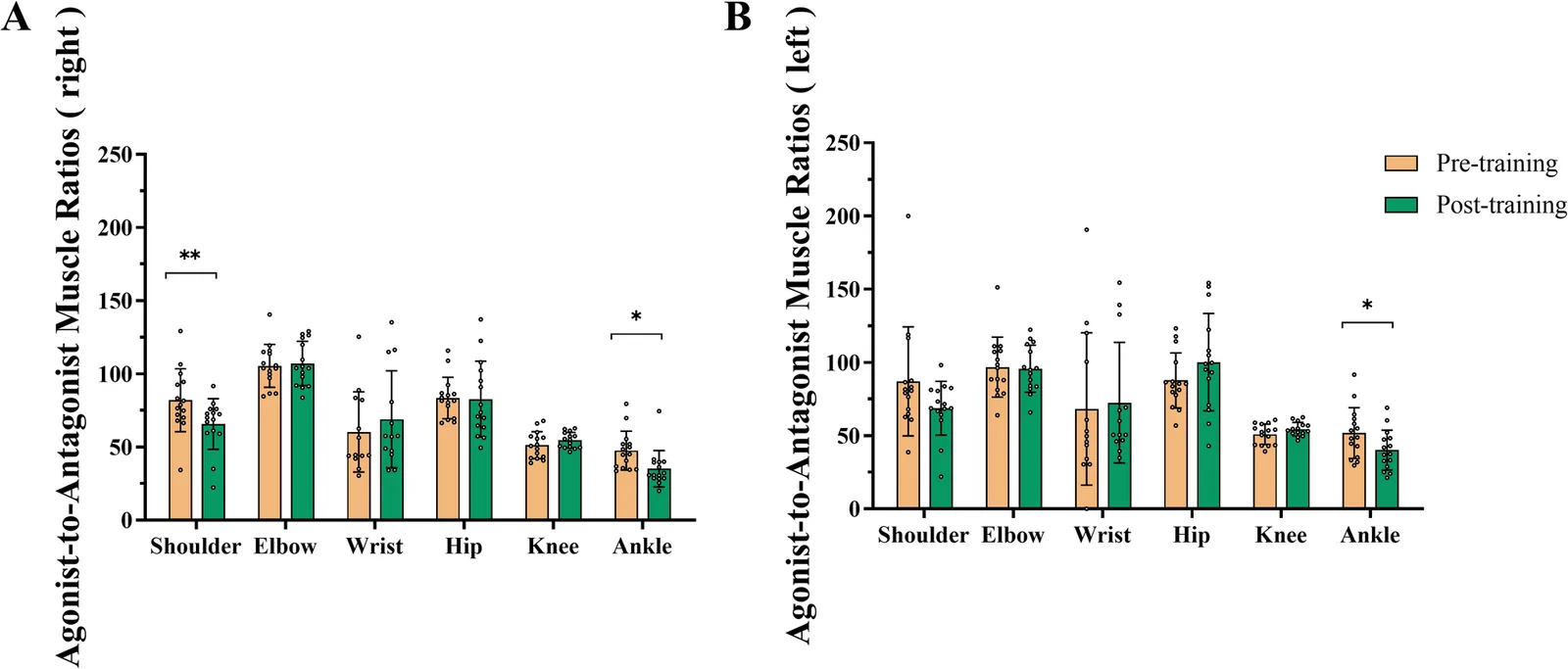
Changes in agonist-to-antagonist muscle ratios (AAR) across joints (n = 15). Repeated-measures ANOVA showed significant reductions in AAR at the right shoulder (p < 0.01) and at both ankle joints (p < 0.05) following training. No significant changes were observed at the other joints. *p < 0.05, **p < 0.01 versus pre-training. Error bars represent 95% confidence intervals

The data are shown in Fig. 6. Repeated-measures ANOVA showed significant gains in plantar flexion. Left ankle plantar flexion PT increased by 37.0% (Cohen’s d ≈ 0.90, *p* < 0.05), and right ankle by 48.6% (Cohen’s d ≈ 1.10, *p* < 0.01). Dorsiflexion parameters showed no significant changes (Fig. 7).

#### Changes in agonist-to-antagonist muscle ratios

 The results in Fig. 7 show the changes in agonist-to-antagonist ratio (AAR) across joints. Repeated-measures ANOVA revealed significant reductions in AAR at the right shoulder (p < 0.01) and at both ankle joints (right: p < 0.05; left: p < 0.05). No significant changes were observed at the other joints (p > 0.05). In summary, significant reductions in AAR were observed at the right shoulder and both ankle joints.

## Discussion

Bone provides essential structural support, protects vital organs, and facilitates movement while adapting to mechanical loading [10, 11]. In this single-arm prospective longitudinal study, 18 months of supervised multicomponent exercise training was associated with significant increases in bone mineral density (BMD) and bone mineral content at multiple skeletal sites — particularly the upper limbs, pelvis, thoracic spine, and whole body — as well as favorable changes in regional lean mass and joint-specific improvements in isokinetic muscle strength among healthy young men with normal-to-high baseline BMD, indicating that these adaptations occurred despite participants already having relatively preserved bone health at baseline. Peak bone mass is typically achieved in the early twenties, and optimizing musculoskeletal health during this critical window may help build greater skeletal reserve for later adulthood and reduce long-term fracture risk [4, 12–14]. The multicomponent training has been proposed as an effective regimen for increasing or preserving bone mass [15–18]. The present findings also suggest that long-term multicomponent training combining resistance, impact, and aerobic elements can produce additional benefits in bone parameters and muscle function even in young men with normal-to-high baseline BMD. Such results are particularly relevant for populations with high habitual activity levels, such as military recruits and athletes, for whom further optimization of bone health and physical performance is desirable. The concurrent increase in fat mass and central fat distribution, despite positive changes in BMD and muscle strength [19], indicates that exercise alone may lead to an overall positive energy balance in this population. This highlights the importance of combining structured training with appropriate nutritional strategies in future interventions. Furthermore, muscle-to-bone ratios decreased across regions, suggesting relatively greater bone adaptation compared with lean tissue.Moreover,our research indicates that multicomponent training can increase the upper-to-lower limb lean soft tissue mass ratio. Therefore,this regional change may serve as a valuable metric for evaluating specific skeletal responses to training and for informing injury prevention strategies [9, 20, 21]. Nevertheless, this positive skeletal adaptation coincides with increased fat mass—a paradox that likely arises because bone responds to local mechanical loading while fat mass reflects systemic energy surplus. Accordingly, the two phenomena are not mutually exclusive but represent distinct physiological domains. The long-term clinical significance of these proportional changes requires further study. However, the results should be interpreted with caution. As a single-arm prospective longitudinal study without a control group, we cannot fully disentangle the effects of the intervention from natural growth-related bone accrual at this age (mean 18.68 years). Dietary intake and habitual activity outside the program were also not strictly controlled. Specifically, the metabolic mechanisms underlying exercise-induced musculoskeletal adaptations remain unclear. Elucidating these mechanisms through future research is critical for informing the design of personalized exercise regimens for optimal health and performance outcomes [15, 22].

In conclusion, this 18-month multicomponent exercise intervention was associated with improvements in several bone parameters and muscle strength in healthy young men with normal-to-high baseline BMD. These preliminary findings also support the potential value of structured training for further optimizing musculoskeletal health even in already active young populations [23, 24]. Future randomized controlled trials with appropriate control groups and integrated dietary monitoring are needed to confirm causality and refine practical recommendations.

### Limitations

This study has several limitations that should be considered when interpreting the results. First, as a single-arm prospective study without a control group, we cannot fully separate the effects of the multicomponent exercise intervention from natural physiological maturation and growth, which are substantial at the participants’ age (mean 18.68 years). Second, although adherence to supervised sessions was high (> 90%), dietary intake, sleep quality, and habitual physical activity outside the program were not strictly controlled. This likely contributed to the observed increases in fat mass and central adiposity. Third, isokinetic strength testing was performed only in a subgroup of 15 participants due to logistical constraints, limiting the generalizability of muscle function findings. Finally, the multicomponent design precludes determination of the independent contribution of each training modality (resistance, impact, or aerobic).

Despite these limitations, the study provides valuable longitudinal data on long-term multicomponent training in young men with normal-to-high baseline BMD. Future randomized controlled trials with dietary monitoring and larger samples are warranted to confirm and extend these findings.

## Conclusions

In healthy young men with normal-to-high baseline BMD, 18 months of supervised multicomponent exercise training was associated with improvements in bone mineral density at multiple skeletal sites and joint-specific muscle strength. These musculoskeletal benefits were, however, accompanied by increases in fat mass and central adiposity. The findings suggest that long-term multicomponent training can further optimize bone health and physical performance even in already active young men with relatively preserved baseline BMD. Nutritional monitoring may be important to optimize the overall response to such programs. Future randomized controlled trials with appropriate control groups are needed to confirm these observations and better define optimal training and nutrition strategies for this population.

## Data Availability

The datasets used and/or analyzed during the current study are available from the corresponding author on reasonable request.
